# Iron bioavailability of a casein-based iron fortificant compared with that of ferrous sulfate in whole milk: a randomized trial with a crossover design in adult women

**DOI:** 10.1093/ajcn/nqz237

**Published:** 2019-10-01

**Authors:** Sharon J Henare, Nadia Nur Singh, Ashling M Ellis, Paul J Moughan, Abby K Thompson, Thomas Walczyk

**Affiliations:** 1 School of Health Sciences, Massey University, Palmerston North, New Zealand; 2 Riddet Institute, Massey University, Palmerston North, New Zealand; 3 NutriTrace@NUS, Department of Chemistry, Faculty of Science, National University of Singapore, Singapore; 4 School of Food and Advanced Technology, Massey University, Palmerston North, New Zealand; 5 Food HQ, The Factory, Palmerston North, New Zealand

**Keywords:** iron, iron absorption, bioavailability, stable isotope, women, dual isotope, erythrocyte

## Abstract

**Background:**

A highly soluble iron–casein complex has been developed for food fortification purposes with the aim to provide high iron bioavailability.

**Objective:**

We aimed to determine the iron bioavailability of the iron–casein complex relative to that of ferrous sulfate (control) when given with whole milk in healthy young women.

**Methods:**

A randomized comparator-controlled trial with a crossover design was conducted using the erythrocyte incorporation dual stable isotope (^57^Fe, ^58^Fe) technique. Iron absorption from the iron–casein complex was compared with that from ferrous sulfate in 21 healthy women aged 20–38 y with normal iron status.

**Results:**

Fractional iron absorption (geometric mean; −SD, +SD) from the iron–casein complex (3.4%; 1.4%, 5.4%) and from ferrous sulfate (3.9%; 1.7%, 6.1%) were not statistically different (*P* > 0.05). The relative bioavailability value of the iron–casein complex to ferrous sulfate was determined to be 0.87 (−1 SD, +1 SD: −0.90, +2.64).

**Conclusions:**

The iron–casein complex has iron bioavailability comparable to that of ferrous sulfate in healthy young women. This trial was registered at www.anzctr.org.au as ACTRN12615000690550.

## Introduction

Maintaining optimal body iron status is important for health and immunity. Diets that are low in bioavailable iron, particularly plant-based diets that contain iron absorption inhibitors such as phytates or polyphenols, can be improved by the inclusion of foods fortified with iron (1, 2). The technological challenge related to producing iron-fortified foods and food ingredients is to identify a form of iron that when added in sufficient quantity provides enough bioavailable iron to meet physiological needs but does not result in adverse physical and sensory changes in the food (3).

Inorganic iron salts are generally used to fortify food products, and these are classified into groups based mainly on their solubility in water. Highly soluble forms of iron are generally the most bioavailable, but they are also the most reactive within food. The interactions between iron and macronutrients in foods can result in oxidation which leads to organoleptic changes (off-flavors). Iron can also cause adverse color changes by reacting with minor food components such as the polyphenolic compounds found in tea, coffee, chocolate, and many fruits (2).

A novel iron fortificant, with iron bound to casein in the presence of orthophosphate and that is highly soluble in water yet stable in food matrixes, has been developed (4–6). Complexes are formed through the interaction of casein, iron, and orthophosphate, with the complexes taking the form of ferric phosphate clusters stabilized in solution by casein molecules (5, 6). Addition of the fortificant in a dairy matrix to beverages results in reduced sensory and color changes compared with other common iron fortificants (4). Because solubility is generally considered a useful indicator of bioavailability, the new fortificant may offer a combination of both high iron bioavailability and stability in foods. As such, it has considerable potential as an iron fortificant for use in liquid milks, yogurts, food powders, and long shelf-life beverages. However, it is important that the bioavailability of iron in the fortificant be evaluated before the new fortificant can be included in foods to enrich diets with iron.

To this end, iron bioavailability of the new fortificant was assessed against that of ferrous sulfate, based on the erythrocyte incorporation of iron from fortificants labeled intrinsically with iron stable isotopes (7) in healthy young women with normal iron status. Ferrous sulfate is widely regarded as the reference (2) for the delivery of iron owing to its high bioavailability compared with other iron sources (8). It was hypothesized, based on relative solubilities, that iron availability between the iron–casein complex and ferrous sulfate would be comparable.

## Methods

### Trial design

A single-blinded, randomized, comparator-controlled trial (ACTRN12615000690550) with a crossover design was conducted between April and May 2015 at Massey University using the erythrocyte incorporation dual stable isotope (^57^Fe, ^58^Fe) method and whole milk as the food matrix. This method compares the iron absorption in 2 liquid meals or fortificants labeled with 2 different iron isotopes. The amount of label absorbed from the liquid meal can be calculated from the shift in the iron isotopic abundances in the blood after RBC incorporation of the absorbed isotopic label, measured 14 d after the administration of the liquid meals (9). Any measurement uncertainty is reduced to <5% when iron absorption efficiency from both meals is expressed relative to each other in the same subject by calculating the relative bioavailability value (RBV). Systematic sources of bias in the individual cancel out and data become comparable between individuals. This refers in particular to iron status as the major regulator of body iron homeostasis by controlling iron uptake by the intestinal mucosa and its release into the circulation. The study protocol was approved by the Massey University Human Ethics Committee (protocol 14/06).

### Sample size

Fractional iron absorption from milk was estimated from the literature to be on the order of 5% for ferrous sulfate for women with normal iron status. Relative differences in iron absorption values >30% between compounds were considered to be nutritionally relevant. Based on data from previous iron absorption studies, the within-subject SD for fractional iron absorption after log10 transformation was 0.14 and the between-subject SD was 0.33. To detect a 30% difference in iron absorption between compounds at an α-level of 5% with a power of 90%, using a 2-sided superiority test, a sample size of *n* = 19 was found to be sufficient.

### Participants

Thirty-six eligible subjects, recruited from the student population at Massey University, Palmerston North, New Zealand, signed informed consent forms and were invited for baseline measurements. Eleven subjects were excluded for not meeting the inclusion criteria and 3 were excluded because they could not participate in the study during the designated time frame. A CONSORT flow diagram showing subjects who were included and excluded is shown in **Supplemental Figure 1**. Subjects were excluded from study participation if they were pregnant, breastfeeding, or if they had any known health problems likely to influence iron status including inflammatory bowel disease, celiac disease, a history of gastric ulcers, disorders of RBCs, menorrhagia, hemorrhoids, hematuria, or malaria. Exclusion criteria also included anemia and allergies to dairy products as well as blood donation or significant blood loss (accident or surgery) within 6 mo before the start of the study, participation in another clinical trial within 3 mo before the start of this study, and former participation in a study involving administration of stable isotopes.

Twenty-two young adult women of Caucasian descent [19–40 y, BMI (kg/m^2^) > 18.5 and < 25] were enrolled in the study by SJH. Venous blood samples were drawn before liquid meal administration for analysis of iron status indexes (hemoglobin and ferritin concentrations) and C-reactive protein (CRP) as an infection marker. All subjects had blood hemoglobin and ferritin concentrations within the reference ranges of 120–160 g/L and 12–150 µg/L, respectively. The CRP values for all participants were <3.0 mg/L, indicating participants were free from infection during the study. Participants were asked to refrain from vitamin/mineral supplement intake or medication, except for oral contraceptives, for 4 wk before the study and until study completion. Subjects were informed about the study orally and in writing. Written informed consent was obtained from all volunteers.

### Isotopic labeling of iron fortificants

All stable isotope–related work and analyses were conducted at the National University of Singapore by staff blinded to the collection of data from the intervention. For all work involving isotopically enriched fortificants and blood samples, working principles of inorganic tracer analysis were followed to minimize sample contamination with natural iron during analysis. Only chemicals of analytical grade or better were used in analyses. Only plastic ware (polyethylene or polypropylene) and Teflon ware (perfluoroalkoxy) were used for analysis and samples were prepared in class 10 laminar flow hoods. Analytical-grade acids were purified by sub-boiling distillation. Isotopically labeled materials were prepared from iron metal (Chemgas), isotopically enriched in ^57^Fe (mean ± SD: 96.7022% ± 0.0066% ^57^Fe) or ^58^Fe (99.887% ± 0.010% ^58^Fe). Isotopically (^58^Fe) enriched ferrous sulfate was prepared by dissolving the isotopically enriched ^58^Fe iron metal in 2 M H_2_SO_4_ (RCI Labscan) and diluting with Milli-Q^®^ water (Millipore, Merck). Aliquots (1.43 mL) of the isotopically labeled [^58^Fe]-ferrous sulfate solution were weighed in individual portions into preweighed PFA vials (Savillex) for administration.

The iron–casein complex (FerriPro) was prepared as described in detail (4–6) with the exception of the use of isotopically labeled (^57^Fe) ferric chloride instead of ferric chloride of natural isotopic composition. The latter was prepared from isotopically enriched ^57^Fe iron metal by dissolution of the metal in 6 M HCl (Merck) and 30% hydrogen peroxide (H_2_O_2_; Merck), which was then concentrated by rotary evaporation. An aqueous solution of sodium caseinate (Alanate 185, Fonterra Cooperative Group Limited) was prepared by stirring 25 g sodium caseinate in 800 mL distilled water at 55–60°C for 2 h. Di-potassium hydrogen orthophosphate (Sigma-Aldrich) was added to the cooled solution to a final concentration of 0.355 M, pH 7.4, and a final volume of 1 L (4–6). This solution was cooled <5°C in an ice bath, and vigorously stirred using a Silverson L4RT high shear mixer while 100 mL 0.5 M labeled ferric chloride (prepared from ^57^Fe as above) was added. The pH was maintained between 6.7 and 6.9 by the addition of 1 M sodium hydroxide (Merck). Stirring continued for another 30 min before heating the solution to 75°C until it became more transparent and reddish-brown in color. The solution was then fed into a laboratory spray dryer (Mini Spray Dryer B-290, BUCHI) at a feed capacity rate of 16–18%, an inlet temperature of 150°C, and outlet temperature of 70°C. A cream-colored powder was obtained that was dispersed in water by vigorous shaking before addition to the liquid meal.

### Liquid meal preparation

Liquid meals (A, B) consisted of 250 mL fresh homogenized pasteurized whole milk (Anchor™ Blue, 3.3% fat, Fonterra Co-operative Group Limited) and 300 mL deionized water. The milk provided 8.25 g protein, 8.25 g total fat, and 12.0 g total carbohydrates per serving. For preparation of solution A, a weighed amount of the isotopically labeled [^57^Fe]-iron–casein complex was dispersed in water on the day of the liquid meal administration. Aliquots of 4.49 mL of the solution containing 2.5 mg Fe/serving were pipetted into the milk ∼1 h before administration. The container containing the dispersed [^57^Fe]-iron–casein complex was shaken thoroughly before pipetting. Emptied glasses were washed twice after milk consumption, first with 50 mL deionized water and then with 40 mL deionized water. The remainder of the deionized water (210 mL) was given to the subjects to drink.

For preparation of solution B, isotopically labeled [^58^Fe]-ferrous sulfate (2.5 mg Fe/serving) was added in aqueous solution to the water, which was given together with the unfortified milk. Vials containing aliquots of 1.43 mL of the labeled solution were emptied into 50 mL deionized water, which was consumed together with the 250 mL of milk (solution B). Direct addition of the labeled ferrous sulfate into the milk would have lowered the pH, resulting in acidification and destabilization of the milk proteins. The emptied glass that had contained the ferrous sulfate solution was washed twice, each time with 20 mL deionized water which was then imbibed. The remainder of the deionized water (210 mL) was given to the subjects to drink. Administered doses of both isotope-labeled iron solutions were determined by weighing vials before and after emptying into the drink. Administered doses varied within ±1% between individuals. Because the 2 iron fortificants were provided to participants in milk (iron–casein complex) or with milk (ferrous sulfate), it was not feasible for all investigators to be blinded to the order in which the participants received the liquid meals. However, investigators were blinded for laboratory analysis of samples and data management.

### Study protocol

Participants were randomly assigned using Research Randomizer computer software (10) to receive the liquid meals in sequence A/B or B/A on consecutive days (*n* = 11 per group). SJH generated the allocation sequence and AKT assigned the participants to the interventions. Body weight and height were recorded before the first liquid meal administration and a venous blood sample was drawn for iron status measurements. On each study day, each subject received 2 liquid meals containing the same isotope-labeled compound (2.5 mg Fe provided by the fortificant per serving) separated by a 3-h interval after an overnight fast, which consisted of abstaining from eating and drinking after 22:00 h until consumption of the first liquid meal. Subjects were asked not to drink or eat between the 2 servings and for 3 h after the second administration. Apart from this, the diet was unrestricted. The alternate iron fortificant was fed on the following day, following the same procedures. All liquid meals were given under close supervision of the investigators. A second blood sample was drawn 14 d after the intake of the last liquid meal for hemoglobin and iron isotopic analyses.

### Preparation of blood samples and fortificants for iron isotope analysis

Samples were prepared for iron isotopic analysis as described previously (11). Briefly, ∼0.2 g blood was mineralized by adding 2 mL 30% H_2_O_2_ (Merck) and 8 mL concentrated sub-boiled nitric acid (HNO_3_) (Merck) using a microwave digestion system (Ethos, Milestone). The digested sample was then dried and redissolved in 6 M HCl for iron separation by ion-exchange chromatography using the strongly basic ion-exchange resin AG 1-X8 (200-400 mesh, DOWEX AG 1-X8, Sigma-Aldrich). The solution was transferred to the top of a column (8 mm inner diameter) filled with the ion-exchange resin to a height of 40 mm. The column was rinsed with 20 mL 6 M HCl after loading and the sample iron was eluted from the column with 10 mL 1 M HNO_3_. The eluate was evaporated to 0.2 mL and then alkalized by the addition of 1 mL 25% ammonium hydroxide (Merck) in an Eppendorf tube. The sample solution was centrifuged at 13790 *x g* for 45 min at 22°C. Precipitated ferric hydroxide [Fe(OH)_3_] was washed twice by Milli-Q^®^ water, each time being centrifuged at 13790 *x g* for 20 min at 22°C. The precipitate was then dried in a 70°C water bath and dissolved in 3 μL hydrogen fluoride (HF). All blood samples were mineralized and analyzed in duplicate (11). Both labeled iron fortificants were prepared for isotopic analysis following the same procedure as described above for blood samples. Fortificant samples destined for iron concentration analysis by isotope dilution MS (IDMS) were mixed with a known mass of a commercially available iron standard (Titrisol^®^, Merck), certified for iron concentration.

### MS

Iron isotope composition of the isotopic labels and the prepared samples was determined by negative thermal ionization MS using FeF_4_^–^ molecular ions and a rhenium double-filament ion source (7, 9). The evaporation filament and the ionization filament were coated with barium fluoride (BaF_2_) to promote the formation of negatively charged ions. The sample iron was loaded as ferric fluoride (FeF_3_) in HF (40%) on top of the BaF_2_ layer on the evaporation filament and coated with a solution of silver nitrate (AgNO_3_) in HF (40%). All mass spectrometric measurements were carried out with a thermal ionization mass spectrometer (Triton) equipped with an array of Faraday cups for simultaneous detection of iron isotope beams. To correct for mass-dependent isotope fractionation effects in the ion source, measured data were normalized to the natural ^56^Fe:^54^Fe ratio.

### Calculation of fractional iron absorption of isotopic labels

Amounts of absorbed iron label were determined from the ratio of circulating isotopic labels to natural iron in blood following principles of IDMS using established algorithms (9). Ratios in blood taken 14 d after liquid meal administration were converted into amounts of absorbed iron based on estimates for blood volume for each individual (12) and an assumed efficiency of incorporation of absorbed label into RBCs of 80% (13). The isotopically labeled iron–caseinate samples were analyzed for iron isotope composition and iron atomic weight as well as iron concentration using reverse IDMS.

### Iron status measurements

Hemoglobin, ferritin, and CRP concentrations were determined by Medlab Central Medical Testing Laboratory (Palmerston North, New Zealand) using standard procedures. Hemoglobin concentration was determined in EDTA-treated blood using the sodium lauryl sulfate method on an automated Sysmex XN20 analyzer. Ferritin concentration was determined in serum samples using an electrochemiluminescence immunoassay (Elecsys^®^ Ferritin, Roche Diagnostics International Ltd) on a Roche Cobas e602 analyzer. CRP concentration was determined in serum samples using the immunoturbidometric method (Roche Diagnostics International Ltd) on a Cobas C analyzer.

### Dissolution tests

Solubility of the iron–casein complex prepared from the [^57^Fe]-ferric chloride as used for the absorption study was compared with batches of iron–casein complex prepared from commercially available ferric chloride hexahydrate (FeCl_3_·6H_2_O; Sigma-Aldrich) of natural isotopic composition. A total of 3 different batches of iron–casein complex were prepared independently to cover batch-to-batch variations. Solubility experiments were conducted twice per batch on different days to cover variations associated with experimental repeatability. Iron content of the different preparations was determined by graphite furnace atomic absorption spectrophotometry (GF-AAS; Varian AA240Z) by external calibration (*n* = 3) using a commercial iron standard (Titrosol^®^, Merck). Samples were mineralized by microwave digestion as described for blood samples. Measurements by GF-AAS were conducted in triplicate.

For solubility testing, ∼250 mL 0.02 M HCl (pH = 1.7; Merck) was added to 200 mg unlabeled iron–casein complex in a beaker with a magnetic stirring bar. The solution was kept at room temperature under constant stirring at 1150 rpm for 2 h which commenced on the addition of acid to the iron–caseinate. Aliquots (1 mL) were pipetted out into microcentrifuge tubes at 5, 10, 20, 30, 45, 60, 90, and 120 min after HCl addition. Each aliquot was centrifuged at 13790 *x g* for 2 min at 22°C, and 900 μL of the supernatant was removed and transferred into another microcentrifuge tube for elemental analysis by GF-AAS. Procedures for the isotopically labeled iron–casein complex were the same but only a single run using a smaller amount (100 mg) could be conducted for solubility testing owing to the limited amount of labeled iron–caseinate available. Solubility at each time point was calculated as the fraction of iron from the iron–casein complex detected in solution taking previous samplings of the solution from the beaker into account.

### Statistical analysis

Statistical analyses were performed using SPSS version 22.0 (SPSS Inc.) and SAS version 9.4 (SAS/STAT). The primary outcome of the study was to determine iron absorption for the [^57^Fe]-iron–casein complex and [^58^Fe]-ferrous sulfate in order to calculate RBV. The differences in iron absorption for the [^57^Fe]-iron–casein complex and [^58^Fe]-ferrous sulfate within subjects were tested for normality and a paired *t* test was used to compare fractional iron absorption. Because the raw data for fractional iron absorption were not normally distributed, the fractional iron absorption values are presented as geometric means. The mean ratio (fractional absorption [^57^Fe]-casein-complex/fractional absorption [^58^Fe]-ferrous-sulfate) was tested for statistical significance from unity (1.0) using a location test. Secondary outcomes of the study were to determine the relations between iron absorption of the [^57^Fe]-iron–casein complex and iron stores (represented by serum ferritin), and between RBV and iron stores. Linear regression analysis on log-transformed data was used to evaluate the relation between iron absorption and iron stores and Spearman's rank-order correlation was used to examine the relation between serum ferritin and RBV. Iron solubility of the unlabeled and labeled iron–casein complexes was tested for normality (Shapiro–Wilk test) and a repeated-measures ANOVA was performed to test the effects of sample and day of testing on iron solubility. Values of *P* < 0.05 were taken as being statistically significant.

## Results

### Iron–casein complex solubility

**FIGURE 1:**
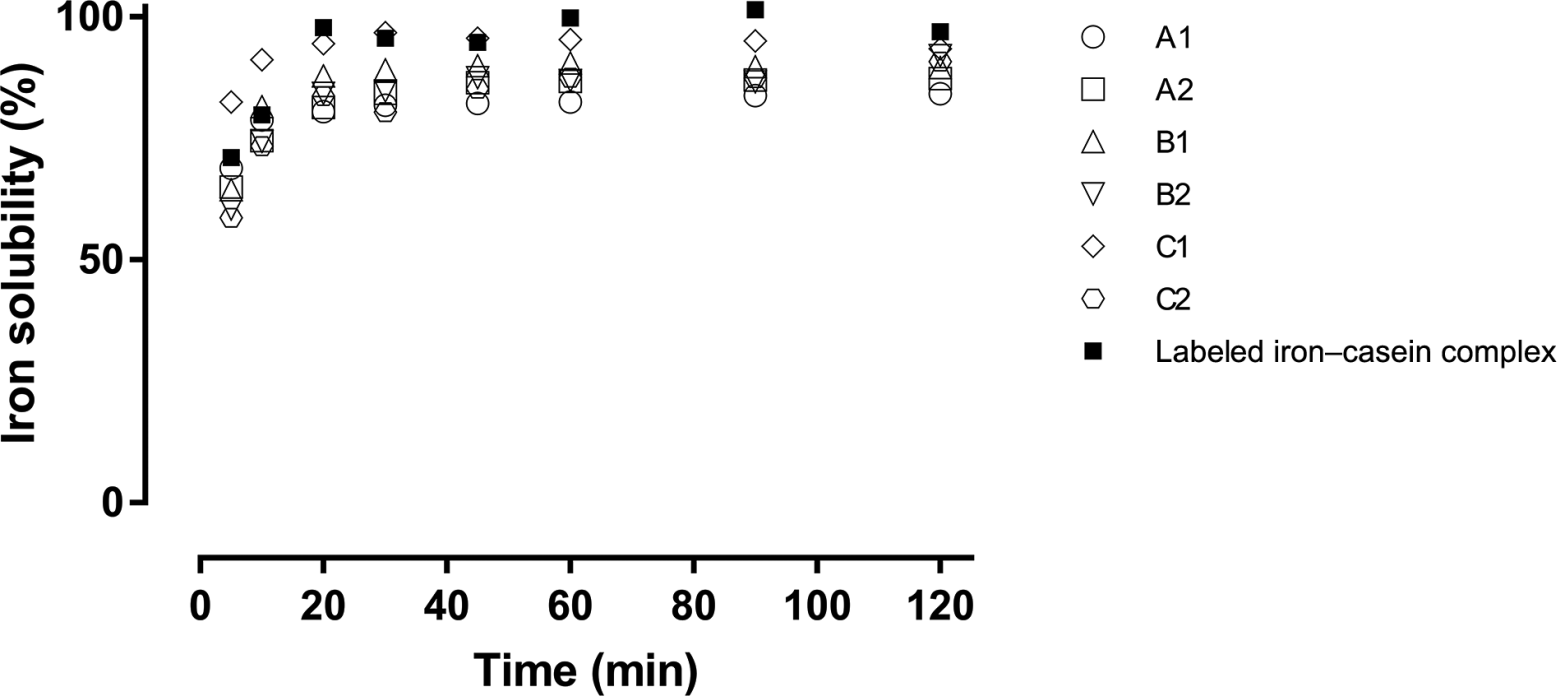
Iron solubility of unlabeled batches (A1, A2, B1, B2, C1, C2) and a labeled (^57^Fe) batch of iron–casein complex in 0.02 M HCl tested on day 1 (A1, B1, C1) and day 2 (A2, B2, C2). There was no statistically significant difference in iron solubility between the unlabeled and labeled iron–casein complexes (repeated-measures ANOVA *F*_3,51_ = 2.13, *P* = 0.11) or between days the samples were tested (*F*_1,51_ = 3.40, *P* = 0.07).

Batches of unlabeled iron–casein complex displayed high iron solubility, similar to that of the labeled counterpart (**Figure 1**). Iron recovery in the aqueous phase reached 60% within 5 min of interaction with 0.02 M HCl. The iron solubility after 2 h ranged from 84% to 96%. Variations (CV) in measured iron solubility of unlabeled iron–casein complex samples ranged from 13% at 5 min to 4% after 2 h between batches and experimental runs. There was no statistically significant difference in iron solubility between the labeled and the unlabeled iron–casein complex (*F*_3,51_ = 2.13, *P* = 0.11) or between the days the samples were tested (*F*_1,51_ = 3.40, *P* = 0.07).

### Bioavailability study

All 22 women completed the study without occurrence of a serious adverse event. The data from one participant were excluded from statistical analysis because her ferritin concentration, which met inclusion criteria at the time of recruitment, was outside the reference range for ferritin (<12 µg/L) on the first day of the study. The characteristics of the remaining 21 subjects are given in **Table 1**.

**FIGURE 2:**
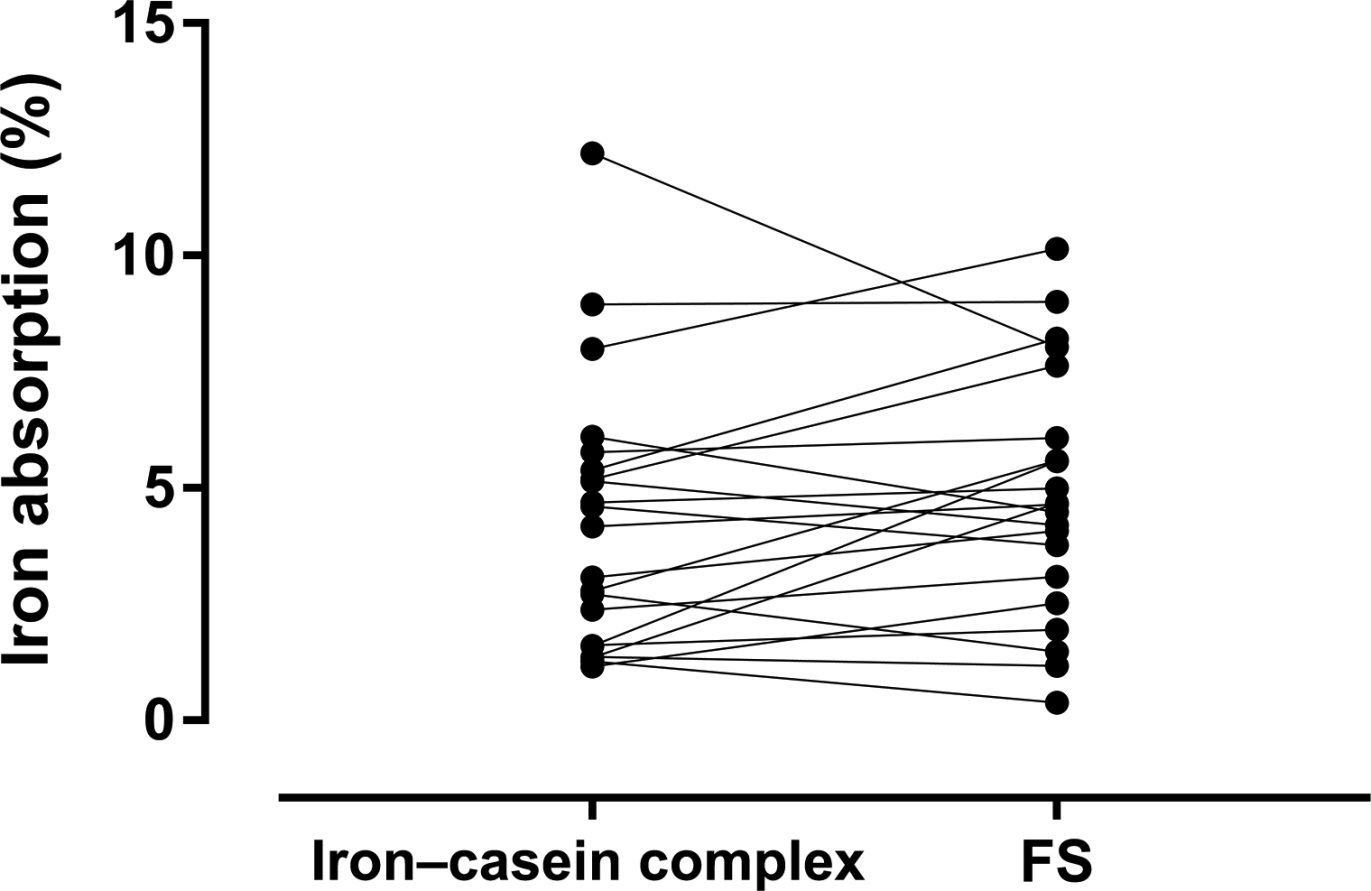
Paired values of fractional iron absorption for young nonanemic women consuming milk with an iron–casein complex or FS. There was no statistically significant difference between fractional iron absorption of the iron–casein complex and FS within subjects (paired *t* test *t* = −1.38, Df = 20, *P* = 0.18). FS, ferrous sulfate.

**TABLE 1:** Baseline characteristics of participants^1^

Characteristic	Value
Age, y	25.2 ± 5.7 (20–38)
Weight, kg	62.7 ± 5.2 (52.6–72.9)
Height, cm	1.70 ± 0.1 (1.6–1.8)
BMI, kg/m^2^	21.8 ± 1.6 (19.2–24.6)
Hemoglobin, g/L	134.7 ± 4.1 (127.0–144.0)
Serum ferritin, µg/L	40.0 ± 2.9 (15.0–72.0)
Serum ferritin,^2^ µg/L	37.85

1 *n* = 21.

2 Values are means ± SDs (ranges) or geometric mean.

Isotope abundances of the labeled [^57^Fe]-iron–casein complex and [^58^Fe]-ferrous sulfate and their final concentrations in the solutions given to the subjects are given in **Table 2**. Fractional iron absorption values for each fortificant are shown in **Figure 2** and the mean fractional iron absorptions in **Table 3**. There was no statistically significant difference between fractional iron absorption of the labeled iron–casein complex and labeled ferrous sulfate (*t* = −1.38, Df = 20, *P* = 0.18). The RBV of the iron–casein complex to ferrous sulfate was determined to be 0.87 (−1 SD, +1 SD: −0.90, 2.64). This ratio was not statistically significantly different from unity (*P* = 0.29).

**TABLE 2:** Isotopic abundances and concentrations of solutions of the [^57^Fe]-iron–casein complex and the [^58^Fe]-ferrous sulfate as fed in the study^1^

	[^57^Fe]-iron–casein complex (*n* = 6)	[^58^Fe]-ferrous sulfate (*n* = 5)
Atomic weight, g/mol	56.94266 ± 0.000014	57.93198 ± 0.00021
Concentration, mol/g	0.00039272 ± 0.00000033	0.0000002948 ± 0.00000022
Concentration, g/g	0.022363 ± 0.000019	0.001708 ± 0.000013
Abundance (%)		
^ 54^Fe	0.00277 ± 0.00042	0.00198 ± 0.00063
^ 56^Fe	1.2777 ± 0.0094	0.0275 ± 0.0090
^ 57^Fe	96.7022 ± 0.0066	0.08394 ± 0.00047
^ 58^Fe	2.0173 ± 0.0042	99.887 ± 0.010

1 Values are means ± SDs.

**TABLE 3:** Fractional iron absorption and the ratio of mean fractional iron absorption of the isotopic labels administered as [^57^Fe]-iron–casein complex and [^58^Fe]-ferrous sulfate in young women^1^

	[^57^Fe]-iron–casein complex	[^58^Fe]-ferrous sulfate	Ratio of mean fractional absorption
Geometric mean	3.41	3.91	0.87
−1 SD, +1 SD of geometric mean	1.39, 5.43	1.72, 6.10	−0.90, 2.64
95% CI of geometric mean	2.48, 4.70	2.74, 5.59	0.59, 1.15

1 *n* = 21. Values are percentages. The ratio of the mean fractional absorption was calculated as the iron absorption of the iron–casein complex/iron absorption of the ferrous sulfate. There was no statistically significant difference between fractional iron absorption of the iron–casein complex and ferrous sulfate within subjects (paired *t* test *t* = −1.38, Df = 20, *P* = 0.18). The ratio was not statistically significantly different from unity (location test *P* = 0.29).

**FIGURE 3:**
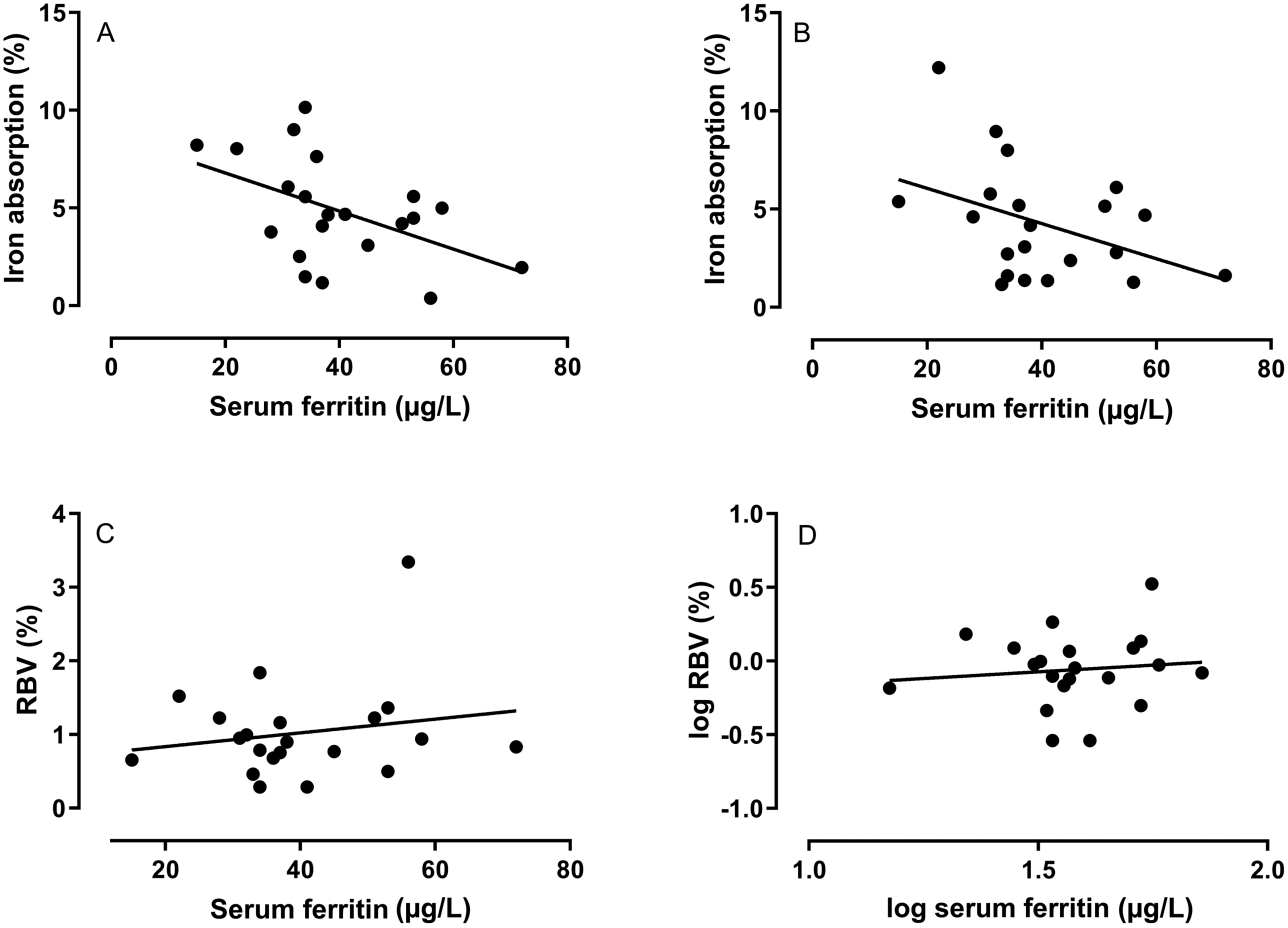
Linear regression relations between serum ferritin and iron absorption from ferrous sulfate (A; *r* = −0.43, *P* = 0.051), serum ferritin and iron absorption from an iron–casein complex (B; *r* = −0.39, *P* = 0.080), serum ferritin and the RBV of ferrous sulfate/iron–casein complex (C; Spearman rank correlation coefficient *r* = 0.04, *P* = 0.850), and log serum ferritin and log RBV of ferrous sulfate/iron–casein complex (D) obtained in young nonanemic women (*n* = 21) who consumed a milk drink containing an iron–casein complex or ferrous sulfate. Regression analyses were performed on log-transformed data. RBV, relative bioavailability value.

There was no statistically significant relation between iron absorption from the iron–casein complex and serum ferritin concentration (*r* = −0.39, *P* = 0.080; **Figure 3**) or between iron absorption from ferrous sulfate and serum ferritin (*r* = −0.43, *P* = 0.051). There was no statistically significant difference between the slopes of the 2 lines (*F*_1,38_ = 0.016, *P* = 0.90). There was no significant correlation between iron stores represented by serum ferritin and RBV (*r* = 0.04, *P* = 0.85).

## Discussion

The aim of this study was to compare fractional iron absorption from a novel iron–casein complex and from ferrous sulfate as a comparator control. Ferrous sulfate is freely soluble in water and is the reference standard widely used in iron bioavailability studies (2). The iron–casein complex is produced by combining aqueous solutions of sodium caseinate and ferric chloride as well as orthophosphate followed by spray drying, which yields a yellowish-white powder as the final product. The resulting compound is a colloidal complex that prevents the precipitation of iron and caseinate in aqueous systems (5, 6). This new technology offers an attractive means of fortifying liquid whole milk, other milk products, and a range of liquid manufactured foods.

Iron deficiency and iron deficiency anemia remain a major public health challenge, particularly among infants, children, and women of childbearing age. Iron fortification of foods is a viable approach to narrow the gap between physiological iron needs and iron supply through the diet. Accordingly, we have conducted our study in healthy, young women as a potential target group at risk of having insufficient iron to balance physiological iron losses including menstrual blood losses. Even a diet that is high in iron may not necessarily provide enough iron for maintaining body function because iron uptake from foods is generally low and highly variable between different foods as well as iron fortificants.

The ideal iron fortificant is highly soluble in water, similar to ferrous sulfate, for the iron to be accessible for intestinal absorption. At the same time, the iron needs to be in a format that prevents it from interacting with dietary compounds that can induce undesirable organoleptic changes and reduce iron bioavailability by formation of insoluble complexes. Both criteria, however, are difficult to meet simultaneously. The high solubility of the iron–casein complex and reduced sensory and color changes when added to beverages (4) suggest the new complex represents a significant technological advancement.

The bioavailability of iron in the iron–casein complex was not statistically significantly different from that of ferrous sulfate when both compounds were consumed along with milk by healthy young women. Whole milk was chosen as the liquid meal because it provides a sufficiently challenging food matrix. Whole milk is known to be technically difficult to fortify with iron. Soluble iron can cause rancidity over time by lipid oxidation, has a distinct metallic taste, and can cause off-flavors or color changes in the presence of polyphenol-containing ingredients such as cocoa powder or when added to breakfast cereals. Milk calcium also has a modest inhibitory effect on iron absorption (14). Ferrous bis-glycine chelate overcomes the inhibition in liquid milk of iron absorption to some extent and is currently used in some countries to fortify liquid milk, dairy products, and multinutrient beverages, having shown good bioavailability (15). However, ferrous bis-glycine chelate is limited in use owing to cost and the potential for promoting organoleptic changes in some food matrixes (15). Less water-soluble iron fortificants such as ferric pyrophosphate (16, 17) or ferric ammonium phosphate (18, 19) cause less adverse effects in milk but at the cost of a lower bioavailability than with highly soluble iron compounds.

Because this research is, as far as we know, the first study evaluating the iron bioavailability of the iron–casein complex, we chose to give the milk without any additional food and drinks other than water as the first step in assessing iron bioavailability of the compound in humans. Previous studies on the iron–casein complex have shown that the complex is less reactive to lipid oxidation in model systems (20). The extent to which iron is protected in the iron–casein complex from interaction with other complexing compounds such as polyphenols or phytic acid that may reduce iron bioavailability will be the subject of future investigations.

Tracer studies are the method of choice for reliable assessment of iron absorption and bioavailability in humans. For evaluation of iron fortificants, however, the tracer must be incorporated chemically into the fortificant to mimic its chemical and physical properties. Tracer iron for this study was acquired in metallic form and converted into [^57^Fe]-ferric-chloride and [^58^Fe]-ferrous-sulfate using standard laboratory procedures. For validation, we compared solubility of the labeled and unlabeled iron–casein complexes at gastric pH as a primary determinant of iron bioavailability. Results (see Figure 1) confirmed the previously reported high solubility of the complex, ranging from 80% to 96% after 30 min with minor increases over the following 90 min. This is in good agreement with the experimental observation of iron absorption being similar between the iron–casein complex and highly soluble ferrous sulfate in our study. Interestingly, some iron remained inaccessible to dissolution even after 2 h with measurable variations between batches. This observation opens up possibilities to improve iron solubility of the complex further by targeted optimization and standardization of the manufacturing process.

In the present study, we compared iron absorption from the iron–casein complex against iron absorption from ferrous sulfate. Whereas the iron–casein complex could be added into the milk before consumption, this was not possible for ferrous sulfate because it would have caused unacceptable organoleptic changes to the milk. We therefore decided to administer the ferrous sulfate in a solution together with the milk. This should have had no effect on iron accessibility for absorption because both the milk and the fortificant would have entered the stomach at the same time. Subjects were randomly assigned to start with either of the 2 liquid meals on the first day of the study followed by the alternate liquid meal on the second day. This controlled for a possible time shift effect, i.e., a difference between 13 d and 14 d after liquid meals for incorporation of absorbed label into RBCs.

Moretti et al. (21) reported that ferrous sulfate was more highly absorbed than micronized dispersible ferric pyrophosphate at low ferritin concentrations but no difference between the fortificants was seen at ferritin concentrations > 40 mu g/L. A similar interaction between ferritin concentrations and RBV was not seen in the current study (Figure 3), and thus it would be expected that the RBV of the iron–casein complex compared with ferrous sulfate would be consistent across individuals of different iron status. As expected, iron absorption was negatively related to serum ferritin concentrations but there was no statistically significant difference between the slopes for the relation, between the 2 fortificants.

Determining RBV instead of measuring absolute iron bioavailability, which is highly variable between individuals and food matrixes, also permits a comparison between findings across studies and different fortificants (see **Table 4**). The RBV for the iron–casein complex was high and comparable with that for ferrous fumarate (8) and micronized dispersible ferric pyrophosphate (17). Ferrous fumarate is highly bioavailable, but it is not suitable for use in a number of food applications owing to undesirable color changes and poor water solubility (16, 22, 23).

**TABLE 4:** RBVs for different iron fortificants compared with ferrous sulfate determined using the erythrocyte dual stable isotope incorporation technique^1^

Compound	Subjects	*n*	Mean ferritin concentration, µg/L	Meal	Fe dose	Ascorbic acid	RBV, %	Reference
Ferrous ammonium phosphate	Young females	19	17.8	Reconstituted milk drink	2.5 mg	√	71	(19)
Ferric pyrophosphate	Young females	19	16.8	Reconstituted milk drink	2.5 mg	√	32	(19)
Ferrous fumarate	Adult females with low iron stores	17	13	Maize and milk drink	4 mg	x	86	(9)
	Adult females	10	16.9	Infant cereal and reconstituted milk	5.0 mg	x	97	(24)
Micronized dispersible ferric pyrophosphate	Young females	10	13.1	Infant cereal and reconstituted milk	5.0 mg	x	62	(21)
	Young females	10	13.1	Infant cereal and reconstituted milk	5.0 mg	√	39	
	Adult females	10	17.1	Infant cereal	5.0 mg	x	82	(17)
		10	26.4	Yogurt drink	5.0 mg	x	93	
Iron–casein complex	Young females	21	37.85	Fresh milk	2.5 mg	x	87	Present study

1 RBV, relative bioavailability value.

In conclusion, the bioavailability of iron from the iron–casein complex was similar to that from ferrous sulfate, as determined in young, nonanemic, healthy women. Our findings suggest that the iron–casein complex offers new possibilities to fortify liquid whole milk and milk products with iron and may address other challenges related to the iron fortification of food.

## Supplementary Material

nqz237_Supplemental_File

## Notes

Supported by the Riddet Institute, Massey University.

Supplemental Figure 1 is available from the “Supplementary data” link in the online posting of the article and from the same link in the online table of contents at https://academic.oup.com/ajcn/.

Data described in the article will be made available on written request and following approval from the Massey University Ethics Committee.

Abbreviations used: CRP, C-reactive protein; GF-AAS, graphite furnace atomic absorption spectrophotometry; IDMS, isotope dilution MS; RBV, relative bioavailability value.
