# Supplementary material for: Iron bioavailability of a casein-based iron fortificant compared with that of ferrous sulfate in whole milk: a randomized trial with a crossover design in adult women
Source: Am J Clin Nutr. 2019 Oct 1;110(6):1362–9. doi: 10.1093/ajcn/nqz237 (PMC6885464; doi:10.1093/ajcn/nqz237)
Supplement: nqz237_Supplemental_File [file nqz237_supplemental_file.pdf]

Supplementary Material

Iron bioavailability of a casein based iron fortificant compared to that of ferrous sulfate in whole milk: a randomized trial with a crossover design in adult women.

SJ Henare, NN Singh, AM Ellis, PJ Moughan, AK Thompson, T Walczyk

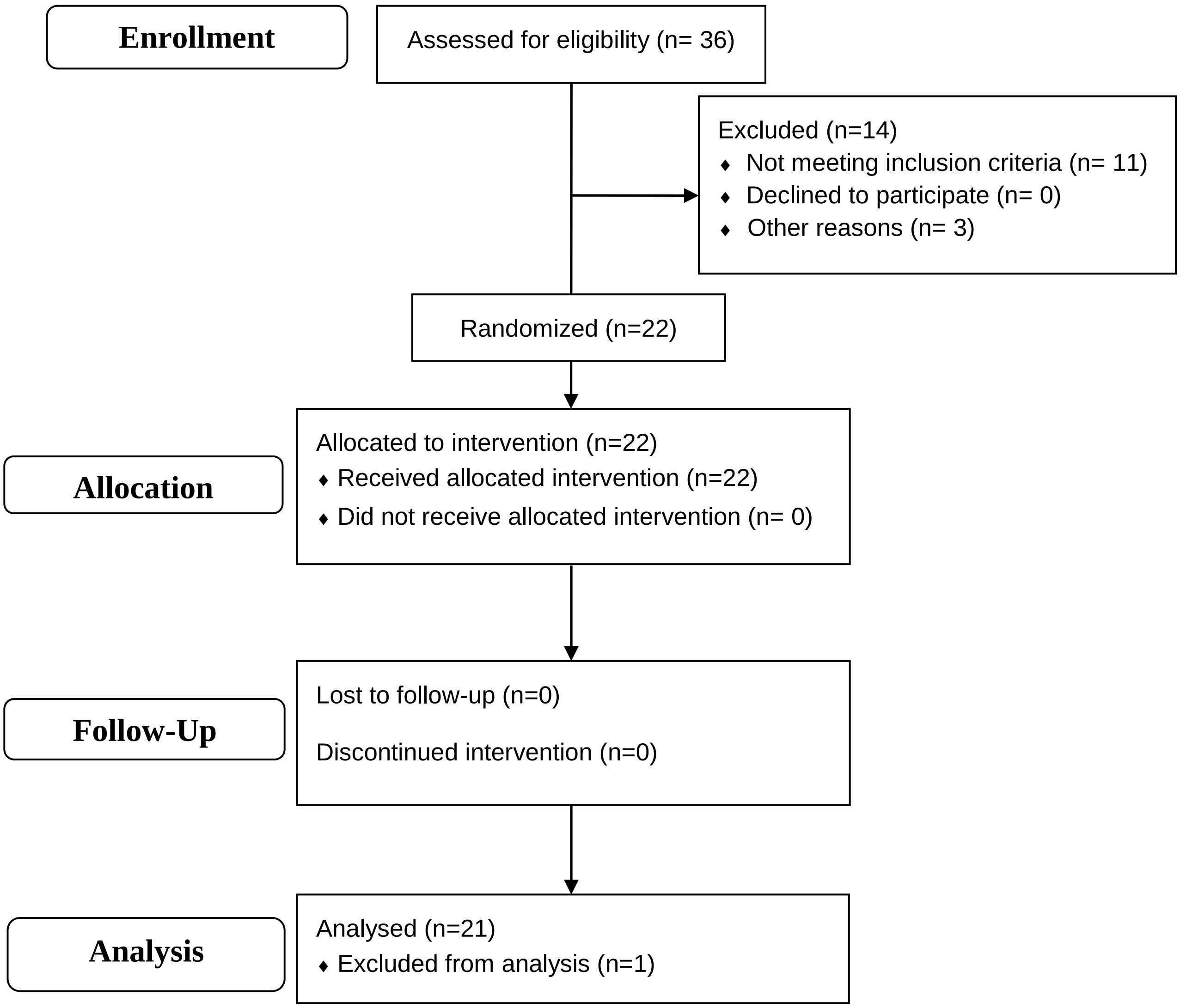

Supplemental Figure 1: CONSORT diagram. Progress of participants through the intervention study. CONSORT, Consolidated Standards of Reporting Trials.

Thirty six eligible subjects were recruited. Eleven subjects were excluded for not meeting the inclusion criteria and three were excluded for not being able to participate during the designated time frame for the study. Twenty two subjects were randomly allocated to receive the intervention. All 22 participants completed the intervention. Data from one subject were excluded from analysis because her ferritin concentration which met inclusion criteria at the time of recruitment was outside the reference range for ferritin (<12 µg/L) on the first day of the study.
